# A Molecular Basis Approach of Eczema and Its Link to Depression and Related Neuropsychiatric Outcomes: A Review

**DOI:** 10.7759/cureus.32639

**Published:** 2022-12-17

**Authors:** Anusha Parisapogu, Blessing T Ojinna, Silpa Choday, Prathima Kampa, Niriksha Ravi, Mingma L Sherpa, Harshita Agrawal, Michael Alfonso

**Affiliations:** 1 Infectious Diseases, Mayo Clinic, Rochester, USA; 2 Internal Medicine and Pediatrics, California Institute of Behavioral Neurosciences & Psychology, Fairfield, USA; 3 Alumna (General Medicine), University of Nigeria Nsukka, College of Medicine, Nsukka, NGA; 4 Internal Medicine, California Institute of Behavioral Neurosciences & Psychology, Fairfield, USA; 5 Internal Medicine and Neurology, California Institute of Behavioral Neurosciences & Psychology, Fairfield, USA; 6 Laboratory Medicine and Pathology, Mayo Clinic, Rochester, USA; 7 Medicine, California Institute of Behavioral Neurosciences & Psychology, Fairfield, USA

**Keywords:** filaggrin, mental health, atopic dermatitis, depression, eczema

## Abstract

"What about my eczema do I love the most? The hurt? A scratch? The humiliation of the public? Oh, there are so many options available!"

Studies have shown an association between atopic eczema (AE), a common inflammatory skin condition, and an increased risk of mental health problems. Despite this, experts are still examining the causes of the links between common mental diseases (such as depression and anxiety) and skin conditions. We collected studies that were published in the past 10 years. We searched the following databases: PubMed, PubMed Central, Science Direct, and Google Scholar. Further relevant research was assessed by examining the bibliographies of eligible studies and related ones. Two reviewers looked at the titles and abstracts of the studies to see if they were eligible, and then they read the full texts.

We went through eczema and depression relationships, their etiopathogenesis, molecular basis, immune response, the role of genetic factors, and possible interactions between neurons and the immune system. Another possible contributing factor could be a change in cutaneous microbiota in eczema patients. Part of the initial connection could be explained by psychological stress, which further leads to depression in eczema patients. Healthcare professionals treating eczema patients must be aware of the comorbidity of mental problems and the potential that people with poor mental health may need social or emotional support. Patients with eczema, especially youngsters, can benefit from routine health checks since they can help identify neuropsychiatric issues like depression early and lessen the burden of both physical sickness and poor mental health. Given that AE is a condition that appears to be related to depression and anxiety, more research with larger samples is needed to determine a potential role for targeted mental health screening in people with AE, as well as the possibility of mental health modification through improved AE control (e.g., using new biologic agents).

## Introduction and background

Skin disorders are among the most frequent human diseases, with eczema being one of the most common [1]. Eczema is a pruritic chronic skin condition characterized by a compromised skin barrier and a strong immune response to environmental stimuli [2]. It's linked to dry skin and intense pruritus [3]. Aetius of Amida was the first who used the expression "eczema" in the sixth century AD [4]. The word "eczema" comes from the Greek word "ekzein," which means "to boil over" or "to erupt." When compared to other chronic medical disorders, it has a high prevalence and a high degree of physical and psychological impact, as well as a lower quality of life [1]. A relationship between dermatologic conditions and neuropsychiatric disorders has been suspected over time. One of the most relevant psychiatric conditions is depression, which is characterized by a loss of interest and pleasure in everyday activities and experiences [3]. Approximately 4.4% of the world's population is affected. Because of the constant itching, discomfort, deformity, and perceived social stigma, people with atopic eczema (AE) seem to be more prone to being depressed [3]. Furthermore, AE-related insomnia may raise the risk of mental disease. In the same line of thought, mental disorders and eczema could affect each other. It has been described that inflammatory mediators in AE may have a role in depression development [3].

About one in nine children and one in fourteen adults have eczema, with the majority suffering from mild diseases [1]. It is a widespread disease that affects 5% to 15% of children and 2% to 4% of adults [2]. According to a recent study, children with eczema in the United States had a much greater incidence of ever experiencing depression (6.5%) and present depression (3.9%) than children without eczema (3.4% in patients with previous depression and 1.8% in patients with present depression). In cross-sectional research that relied on self-reported exposures and outcomes, AE has been linked to common mental disorders (depression and anxiety) and suicidality [5]. The severity of symptoms of eczema can be influenced not only by biological variables such as irritants and allergens but also by psychosocial factors such as stress [6]. Psychological stress is frequently mentioned as a contributing factor in AE [7]. However, the link between eczema and depression in adults and children remains under study [2].

The hypothalamic-pituitary-adrenal axis is activated by stress, resulting in higher amounts of T helper and natural killer cells. T cells have an important role in the etiology of AE. A thorough observational analysis of longitudinal patient data [8] was conducted and it has been determined that persistent eczema increases a patient's chance of suicidal death. However, there is a paucity of clinical information linking stress to these diseases [7]. Psychosocial factors may impact the intensity of AE symptoms through behavioral or psycho-neuroimmunological mechanisms [6]. Complex psycho-neuro-immunological pathways are thought to play a significant role in the development and progression of the disease, particularly in adults. It has been observed that educational interventions can help with AE management [6]. From a global viewpoint, further research is required to understand completely eczema's psychological co-morbidity. Longitudinal data is limited and contradictory. The exact timing of any link between AE and depression is unknown. As a result, this review aims to look into the relationship between psychosocial variables and depression in eczema patients and to assess how depression and eczema interact with each other.

## Review

Methods

Data was gathered from the following databases: PubMed, Medline, PubMed Central (PMC), and Google Scholar. We searched for articles that were relevant to eczema and depression. We conducted the literature search by using the keywords: eczema, depression, atopic dermatitis, and related terms. We found 817 articles altogether that are relevant and peer-reviewed, and duplicates were removed. We screened titles and abstracts, which led to 370 articles and finalized 52 articles in total as our references. All the studies that are included were published in English within the last 10 years of the search date. We have not considered any specific geographical location for the study search. Our study results included both the abstract and full-text articles.

Results

We focused on the potential etiopathogenesis of eczema, its molecular mechanism, role of shared cytokines in depression and eczema. We also went through the interactions between the neurons and the immune system and the role of psychological stress which is further leading to depression in eczema patients. All the data was collected ethically and legally.

Discussion

The etiopathogenesis of AE is still under study. Three dimensions appear to be important, including epithelial barrier dysfunction, immune response, and interactions between neurons and the immune system.

Epithelial Barrier Dysfunction

The stratum corneum is made up of vertical layers of keratin filament-filled anucleate corneocytes [5,9] and regulates the homeostasis of water and serves as the first line of defense against environmental allergens and infections. Due to the altered stratum corneum, there is an increase in transepidermal water loss, which results in increased permeability, decreased water retention, and altered lipid composition [10,11]. It is primarily here that the epidermal barrier function is found [5,9]. However, the fundamental abnormality in the onset of atopic dermatitis is epidermal barrier failure [11].

Role of filaggrin (FLG) gene: The FLG gene encodes the filaggrin protein, which is situated on chromosome 1q21 in the epidermal differentiation complex (precursor profilaggrin). It's one of the natural moisturizing factors (NMF) components [12]. In low-humidity environments, skin moisture and water retention in the stratum corneum are preserved by NMF [12]. The main genetic risk factor in atopic dermatitis is FLG loss-of-function mutations [13]. FLG loss-of-function mutations have been linked to a three- to fourfold higher risk of developing atopic dermatitis in meta-analyses [14-16]. Thirty-one loci associated with atopic dermatitis were revealed in a meta-analysis of 26 genome-wide association studies (GWAS) comprising approximately 21,000 cases and 95,000 controls [17].

Other factors that can lead to skin barrier breakdown include protease and antiprotease activity in the stratum corneum are out of balance in the stratum corneum. Faulty tight connections might compromise the skin barrier. Claudins, Junctional adhesion molecule A (JAM-A), occludin, and tricellulin are transmembrane proteins that make up tight junctions. The claudin-1 expression has been observed to be reduced in atopic dermatitis patients' non-lesional skin [18,19]. Microbial colonization and the generation of proinflammatory cytokines [20]. Filaggrin synthesis in the skin is inhibited by inflammatory cytokines, including interleukin (IL) 4 and others including IL-13, IL-17A, IL-22, IL-25, and IL-31, causing further damage to the barrier [21].

Immune Response

Both innate and acquired immune responses contribute to the pathogenesis of type 2 inflammation in atopic dermatitis [22,23]. When bacteria or tissue damage activate innate immune receptors like Toll-like receptors (TLRs), several signals (alarmins), and extracellular matrix (ECM) proteins including periostin are released [24]. Alarmins are released when the epithelial barrier ruptures, activating T helper 2 (Th2) cells, skin-resident group 2 innate lymphoid cells (ILC2s), mast cells, and basophils, as well as inflammatory dendritic epidermal cells and type 2 immune cells. IL-4 and IL-13 are released by activated Th2 cells which increases inflammatory response and B cell immunoglobulin E (IgE) class switching, which activates the STAT (signal transducers and activators of transcription) pathway to produce antigen-specific IgE molecules [25]. Th2 cytokines like IL-4, IL-13, IL-31, and IL-22 decrease the expression of genes involved in terminal keratinocyte differentiation (e.g., FLG, loricrin, and involucrin), prevent the production of adenosine monophosphates (AMPs) and encourage epidermal hyperplasia in addition to their role in increasing inflammation [26,27].

Interactions Between Immune System and Neurons

Atopic dermatitis is characterized by chronic itch. Itch is caused by the transmission of signals from primary sensory neurons' (pruriceptors') cell bodies in the dorsal root ganglia to peripheral C-nerve fibers that are unmyelinated, histamine-sensitive, and non-histamine-sensitive [27]. Histamine, cytokines, proteases, and other endogenous and exogenous irritants, as well as their receptors, stimulate nerve terminals in the epidermis, papillary dermis, and skin appendages [27]. Chronic itch is caused by complex interactions between non-histamine-sensitive peripheral C-nerve fibers, Th2 immune cells, and keratinocytes in atopic dermatitis, and type 2 cytokines are hypothesized to be important mediators of chronic itch [28]. The importance of these neuroimmune interactions in the pathophysiology of chronic atopic itch is supported by the itch response to duplilumab and Janus kinase (JAK) inhibitors, which disrupt IL-4 signaling and IL-4 receptor inhibition, respectively [29].

**Figure 1:**
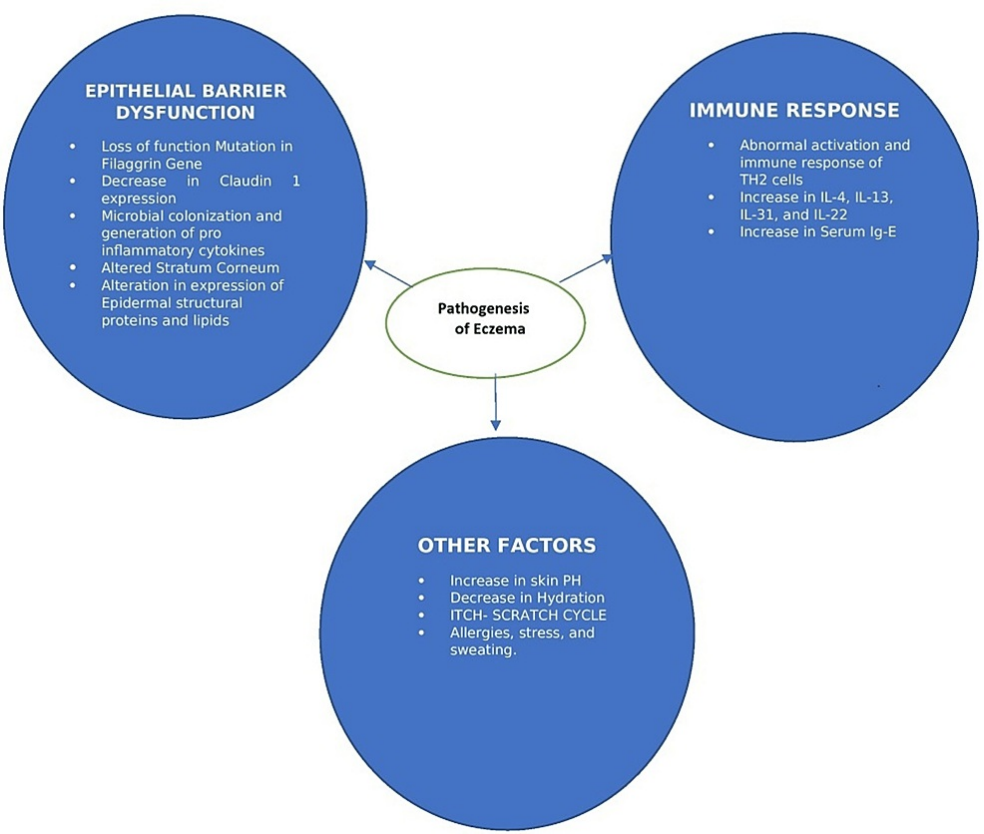
Dimensions of the Pathogenesis of Eczema TH2: T helper type 2, IL: interleukin, Ig: immunoglobulin, PH: potential of hydrogen

*Staphylococcus aureus (S. aureus)* overgrowth and reduced bacterial diversity were reported to be major changes in the skin microbiome of most people with atopic dermatitis, especially in lesional skin [30]. According to a meta-analysis of 95 observational studies, 70% of individuals with atopic dermatitis had S. aureus on lesional skin (95% Confidence Interval (CI) 66-74), while 39% had S. aureus on nonlesional skin (95% CI 31-47) [31]. The following Figure 1 summarizes the dimensions of the pathogenesis of eczema.

Role of cytokines in depression: Inflammatory mediators in AE may have a role in depression [3]. Numerous symptoms, including fatigue, are shared by somatic diseases and depression. This suggests that the activation of the cytokine system may contribute to the development of depressive symptoms and sick behavior in the majority of somatic diseases, including eczema [32,33]. Additionally, according to the "cytokine hypothesis of depression," cytokines play a crucial role in the central behavioral, neurochemical, and neuroendocrine mediation that are aspects of depressive disorders [33]. Over recent years, it has been established those pro-inflammatory cytokines such as IL-1, and IL-6, along with tumor necrosis factor-a (TNF-a), induce true major depressive disorders in physically ill patients with no prior history of mental disorders along with illness symptoms [34]. For instance, when depressed female patients are compared to healthy female patients, TNF-a levels are higher [35]. Additionally, it has been hypothesized that the immunological modifications linked to depression may be a factor in the pathophysiologic processes underlying dyslipidemia, osteoporosis, and diabetes [35].

The hypothesis that inflammatory cytokines are causally involved in behavioral abnormalities in people with depressive disorders was confirmed by experiments involving immunological stimulation in humans [36,37], which showed that immune stimulation produces signs and symptoms of depression. Additionally, there is evidence that depressive symptoms and fatigue are caused by cytokines in inflammatory cerebral diseases such as multiple sclerosis [38]. TNF-a is a part of the cytokine system, which has been linked to the etiology of depression by three different mechanisms.

Initially, several authors hypothesized that cytokines would possibly stimulate neuronal serotonin transporters since proinflammatory cytokines and serotonergic homeostasis were both associated with the etiology of significant psychiatric illnesses [39]. As selective serotonin reuptake inhibitors (SSRIs) aid in depression recovery by serotonin transporter deactivation, this proposal would confirm the hypothesis of a serotonin deficit in depression and the pharmacodynamic mechanism of SSRIs in the treatment of depression [39]. Increased pro-inflammatory cytokine production activates the tryptophan- and serotonin-degrading enzyme indolamine-2,3-dioxygenase (IDO), which links cytokine activation. Increased consumption of serotonin and its precursor tryptophan as a result of IDO activation may explain why serotonin availability is reduced in depression. Proinflammatory cytokines activate IDO, resulting in the generation of glutamatergic agonists [40,41]. And the third mechanism says that it has been suggested that the activation of cytokines may be a cause of the activation of the hypothalamic-pituitary-adrenal (HPA) system due to depression [42].

Role of psychological stress in eczema causing depression: A variety of theories have been advanced to clarify the mechanisms behind or contributing to the association between atopic diseases and mental illnesses. Some of these factors, such as stress from poor family functioning or even traumatic life events, may have a role in both diseases [42]. In the study done by Buske-Kirschbaum et al., attention deficit hyperactivity disorder (ADHD) raises stress levels, which can stimulate neuroimmune pathways that contribute to eczema [43]. Many studies have found that psychosocial stress is linked to the intensity of symptoms in AE patients. Hill's criteria for causality state that the relationship between stress and the severity of AE is weak to moderate based on the correlation coefficients found in studies [25]. The association is not very specific in terms of specificity. However, this isn't always a justification for non-causal inference [25]. A few longitudinal studies' findings that there is a relationship between timing and stress suggest that stress can be assessed before symptoms of higher intensity start to appear.

Due to expensive and time-consuming treatments, eczema can generate psychological stress, but stress also seems to make symptoms worse. As a result, a vicious loop could develop as a result [25]. They were unable to draw any conclusions about the biological gradient or dose dependency of stress. Because stress appraisal is subjective, measuring the dose of stress is difficult [25]. The following table summarizes the studies selected for the pathogenesis and molecular basis of eczema (Table 1).

**Table 1:** Pathogenesis and the Molecular Basis of Eczema TEWL: transepidermal water loss, FLG: filaggrin, AD: atopic dermatitis, CLDN-1: Claudin-1, PCR: polymerase chain reaction, SNP: single nucleotide protein, TH2: T helper 2 cells, mRNA: messenger RNA, TJ: tight junction

Author (Year)	Purpose of Study	Study Characteristics	Outcomes and Results	Conclusions
Kelleher M et al., 2015 [10]	To see if a noninvasive evaluation of skin barrier function on the second day after birth and at two months predicts the onset of Atopic Dermatitis at one year. In addition, researchers wanted to see if increases in transepidermal water loss (TEWL) preceded the onset of clinical Atopic Dermatitis.	From July 2009 to October 2011, a total of 1903 newborns were enrolled in the Cork Babies After Scope: Evaluating the Longitudinal Impact Using Neurological and Nutritional Endpoints Birth Cohort study. The TEWL was measured at birth (day 2), 2 months, and 6 months. At 6 and 12 months, the presence of AD was confirmed, and the severity of the condition was determined using the Scoring Atopic Dermatitis clinical instrument at 6 months and 12 months. A total of 1300 newborns had their FLG mutations genotyped.	18.7% of the children had AD at 6 months, and 15.53% at 12 months. In a logistic regression model, the day 2 upper quartile TEWL measurement was strongly predictive of AD at 12 months (area under the receiver operating characteristic curve, 0.81; P.05).	Clinical Atopic Dermatitis is preceded by a loss of skin barrier function at birth and at two months. These findings have implications for the best timing of therapies for the prevention of Atopic Dermatitis, in addition to offering crucial mechanistic insights into disease development.
Rodriguez E et al., 2009 [15]	To better refine FLG risk profiles within the broad and inclusive eczema diagnosis and to provide a more accurate estimation of FLG effect magnitude and also to get a more precise and clear estimation of the asthma risk associated with FLG null alleles.	meta-analysis	strong associations with eczema	FLG mutations confer a high eczema risk, and their risk profiles are refined, revealing a link with more severe diseases. Additionally, a significant risk factor for asthma, FLG mutations could help define the asthma endophenotype that is associated with eczema.
De Benedetto A et al., 2011 [19]	screened two American populations for single nucleotide polymorphisms in the claudin-1 gene and examined the expression/function of the TJ protein claudin-1 in epithelium from AD and nonatopic people (CLDN1).	Illumina's BeadChips were used to produce expression profiles of nonlesional epithelium from extrinsic AD patients, nonatopic individuals, and psoriasis patients. The use of tissue staining and quantitative PCR to confirm dysregulated intercellular proteins was used. In Using chambers, epithelial bioelectric characteristics were determined. Using a knockdown technique in primary human keratinocytes, the functional importance of claudin-1 was examined. In two separate groups with Atopic dermatitis, 27 haplotype-tagging SNPs in CLDN1 were examined.	Only patients with Atopic dermatitis had significantly lower expression of the TJ proteins claudin-1 and claudin-23, which was confirmed at the mRNA and protein levels. The expression of Claudin-1 was found to be inversely associated with TH2 biomarkers. They discovered a significant deterioration in the bioelectric barrier function in the epidermis of those with Atopic Dermatitis. They found that inhibiting claudin-1 expression in human keratinocytes reduces TJ function while increasing keratinocyte proliferation in vitro. Finally, CLDN1 haplotype-tagging SNPs in two North American groups found correlations with Atopic dermatitis.	Tight junction dysfunction contributes to barrier failure and immunological dysregulation in Atopic Dermatitis patients, and this may be mediated in part by claudin-1 decreases.

Depression and eczema in the clinical scenario

In a cross-sectional study, despite the modest odds ratio, eczema patients had a considerably increased rate of depression [44]. In the study, of 188,495 participants, in several locations, they found a strong positive link between eczema and depression risk. A Norwegian study found that young people with eczema and itchiness had a 24% higher frequency of suicidal thoughts in the week prior [1] and eczema has also been associated with poor mental health. An analysis of data from the 2007 National Survey of Children's Health in the United States found that the prevalence of depression and anxiety disorders among children with atopic dermatitis was significantly higher than among their peers without atopic dermatitis and also showed an upward trend with increasing parent- and caregiver-reported dermatitis severity [45].

More than 8000 adults and adolescents with atopic dermatitis and age- and sex-matched controls were included in a large, longitudinal cohort study using data from the Taiwan National Health Insurance Research Database from 1998 to 2008 to determine the likelihood of developing a major depressive disorder or anxiety disorders later in life [46]. This study [46] found that atopic dermatitis patients had a higher incidence of major depression, any depressive disorder, and anxiety disorders. When it comes to long-term effects, the burden of disease is typically significant for individual patients due to discomfort or itching, sleep difficulties, feelings of stigmatization, restrictions on leisure activities, prolonged sick leave from work or school, reduced social contact, and time-consuming therapy, to name a few [47]. According to the Social Readjustment Rating Scale, partner bereavement is perceived as one of the most stressful acute life events. It impacts most people negatively [7]. Still, that impact could be observed more in people with eczema.

Atopic disease and psychological risk factors in the pediatric population

In a cross-sectional child health survey done in Copenhagen in the autumn of 2009, comprising 9215 individuals who possessed at least one atopic disease, one mental health issue, and socio-economic factors, it was concluded that emotional difficulties were slightly higher in girls than in boys, but hyperactivity problems were higher in boys than in girls. Emotional difficulties rose with age, but conduct problems, hyperactivity problems, and peer problems were higher in children aged 15. All problem scores increased when the primary caregiver's educational level and household income decreased [48]. Additionally, it was found that in children, symptoms like behavioral disturbances and hyperactivity difficulties were marginally greater than with any known atopic condition ever or with current symptoms. Children with current symptoms of the relevant atopic condition had higher levels of hyperactivity problems, emotional problems, and slightly higher levels of conduct problems than children without current symptoms of the atopic condition, even after controlling for age, gender, and the educational level of the primary caregiver. Additionally, they found significant differences in mental health issues based on socioeconomic status.

When comparing children with little education to children with high education, the link between present asthma symptoms and hyperactivity problems was higher [48]. In contrast to previous findings, a Danish study showed no differences in child-reported overall mental well-being or parent-reported peer problems when comparing children aged 6-9 years with and without registered information on doctor-diagnosed asthma or allergies [40]. Two cross-sectional studies of children aged 5-15 years and 14-16 years found an increased risk of hyperactive and emotional disorders among children with asthma, whereas only one of these revealed a higher risk of conduct problems and only one found no association with peer difficulties [41,49].

In an American cross-sectional study of children aged 0-17 years, a history of eczema was linked to increased risks of ADHD, anxiety, depression, and conduct disorder [45]. However, two Korean studies, contrary to many findings, revealed no link between internalizing behaviors and eczema or externalizing behaviors and any of the behavioral disorders. This could be due to their small sample sizes, a lack of correction for possible confounders, or cultural differences between Korea and other countries [50,51]. Parental mental health problems increase the likelihood of smoking and may cause atopic disease in children who are already predisposed to mental health issues [52].

The following table summarizes the studies selected for psychological associations with Eczema (Table 2).

**Table 2:** The Psychological Associations with Eczema AE: atopic eczema, AR: atopic rhinitis, RR: relative risk

Author (Year)	Purpose of Study	Type of Study	Outcomes and Results	Conclusions
Drucker AM et al., 2018 [1]	To determine the link between eczema and a patient's later risk of suicide death.	Double-matched case-control study.	The connection between eczema and suicide death was estimated using logistic regression. Chronic eczema is linked to a higher risk of suicide.	Patients with persistent eczema were at a slightly increased risk of suicide in the future, despite the fact that this is dependent on the patient's general mental health and the absolute risk is low. Physicians who care for these individuals have the opportunity to intervene to prevent suicide.
Bao Q et al., 2018 [2]	To look into the link between eczema and the risk of depression.	Systematic review	The pooled RR for eczema and depression risk was 2.02 (95% CI 1.76 to 2.31, I2 = 33.7%).	Patients with eczema were found to have a higher risk of depression. According to these findings, clinical practitioners should continue to be mindful of the link between eczema and the risk of depression.
Schonmann Y et al., 2022 [3]	The purpose of this study was to look at the relationship between atopic eczema and new depression/anxiety.	Matched cohort study	Atopic eczema was linked to a higher rate of new depression and anxiety.	Adults with atopic eczema are more likely to experience anxiety and sadness. There was a dose-response association between depression and the severity of atopic eczema.
Ring J et al., 2019 [4]	To shed light on the genuine pain and individual burden of disease experienced by adult AE patients: Between October 2017 and March 2018, experienced interviewers conducted a computer-assisted telephone interview. The Patient-Oriented Eczema Measure (POEM), Dermatology Life Quality Index (DLQI), Hospital Anxiety and Depression Scale (HADS-D), and a newly designed Atopic Eczema Score of Emotional Consequences were used to assess the severity and measure the quality of life (AESEC). Patients were also asked to rate the severity of their illness on a scale of one to ten.	Observational study	When asked how they felt about their eczema, 57% said they were emotionally burdened by feelings like "trying to hide eczema," "feeling guilty about eczema," "issues with relationships," and more. Eighty-eight percent of people with severe AE reported that their condition had hampered their ability to face life in some way.	Adults with a moderate to severe type of AE suffer more than would be considered tolerable, according to this real-life study.
Yaghmaie P et al., 2013 [45]	aimed to determine the mental health burden linked with Atopic Dermatitis in children in the United States.	Cross-sectional study	The incidence of a mental health issue and the reported severity of the skin illness had a clear dose-dependent connection.	In the US pediatric population, there is a strong link between mental health issues and Atopic Dermatitis. The strength of the link is affected by the degree of skin illness.
Chang HY et al., 2013 [50]	Using data from a community survey, researchers looked into the link between three common allergic disorders: asthma, allergic rhinitis (AR), atopic dermatitis, and psychological and behavioral issues in preschoolers.	Cross-sectional survey	In the study, those diagnosed with AR had considerably higher internalizing and sleep difficulties scores. Attention issues and attention-deficit/hyperactivity disorder scores were greater in preschoolers who had been treated for atopic dermatitis in the previous 12 months. According to the Atopic Dermatitis Scoring Index, sleep problems were more severe in moderate-to-severe Alzheimer's disease patients than in control and mild Alzheimer's disease patients. It was found that the number of eosinophils in peripheral blood was strongly linked to how hard it was to sleep.	The three major allergy disorders have different psychological and behavioral issues, with asthma having a weaker link and AR and AD having a stronger association. The findings of this study could lead to the discovery of common underlying processes in allergy disorders as well as psychological and behavioral-related issues.
Park J et al., 2011 [52]	To look at the link between psychosocial aspects and symptoms of allergy illnesses, as well as the link between behavioral issues and atopy biomarkers.	Cross-sectional survey	Children with asthmatic symptoms had significantly more externalizing problems, while children with both asthma and allergic rhinitis symptoms had significantly more internalizing problems. Children with allergic rhinitis and atopic dermatitis had much worse social adjustments. Boys who tested positive for more allergens via skin prick testing had greater internalizing issues.	While it has been documented that school students with allergic symptoms have more difficulty with psychosocial adaptation, the patterns of psychosocial problems vary depending on the type of atopic illness. There was a link between atopy and behavioral issues, particularly in boys.

## Conclusions

Giving specific attention to the treatment of psychological aspects may help patients with their skin conditions and enhance their quality of life. Cytokines and neurohumoral impact are related to the cause of this association as well as the mechanism that underlies it. First, psychiatric disorders like depression may arise as a side effect of the main skin issue, as a reaction to social shame, physical deformity, or negative lifestyle changes caused by the condition. Second, illness disruption attachment may result in psychological difficulties and possibly raise the likelihood of depression in adult patients. Additionally, a vicious cycle supported by an inflammatory cytokine response has been shown to link depression with eczema. When treating eczema patients, healthcare providers must be aware of the comorbidity with mental disorders as well as the possibility of poor mental health might require social or emotional support. Regular health examinations of eczema patients, especially children, can aid in the early detection of neuropsychiatric problems like depression and decrease the burden of both physical illness and poor mental health. More research needs to be done to find out what causes neurohumoral inflammation and how important it is for long-term follow-up, prevention, and treatment.
